# Thyrotoxic Crisis in the Absence of Risk Factors: A Case Report

**DOI:** 10.7759/cureus.39972

**Published:** 2023-06-05

**Authors:** Gowry Reddy, Jonathan Livingston, Darshan Gandhi, Fadwa Sumrein

**Affiliations:** 1 Internal Medicine, New York Medical College at St. Mary's General Hospital, Passaic, USA; 2 Internal Medicine, New York Medical College at St. Clare's Health, Denville, USA; 3 Internal Medicine, New York Medical College at St. Mary’s General Hospital, Passaic, USA; 4 Internal Medicine, New York Medical College at St. Clare’s Health, Denville, USA; 5 Endocrinology, St. Mary's General Hospital, Passaic, USA

**Keywords:** graves' disease, thyroid storm, hyperthyroid, thyroidectomy, goiter, thyrotoxic crisis

## Abstract

Thyrotoxic crisis is a severe, life-threatening form of thyrotoxicosis characterized by elevated circulating thyroid hormone that can lead to profound complications. Early diagnostic interventions include a thorough physical examination, laboratory assessments of thyroid hormone levels, and the utilization of quantifying assessment tools to grade the severity of the condition. A targeted therapeutic regimen involving a combination of thioamides, beta-blocking agents, and iodide therapies is administered to combat each stage of the physiological process involved in a thyroid storm. The prompt recognition of clinical manifestations and systemic complications of thyrotoxic crisis is of paramount significance to prevent therapeutic delay and reduce patient mortality. Here, we report an atypical case of a new-onset thyrotoxic crisis in a patient without apparent underlying predisposing factors.

## Introduction

Thyrotoxic crisis is an acute and severe form of thyrotoxicosis. It is a rare clinical entity involving excess thyroid hormone activity secondary to high levels of circulating thyroid hormones in the body. It occurs in approximately 2% of women and 0.2% in men [1]. The mortality rate of thyrotoxicosis is estimated to be approximately 8-25% in all cases. It accounts for nearly 1-2% of hospital admissions with a breakdown of 4.8 to 5.6 cases per 100,000 per year in hospitalized patients [2]. The clinical presentation of thyrotoxicosis encompasses a broad spectrum of symptoms ranging from an asymptomatic state to a life-threatening thyroid storm. Common etiologies often include Graves’ disease, toxic multinodular goiter, and toxic adenoma, in addition to thyroiditis, drug-induced and factitious hyperthyroidism, exogenous hormone replacement, and acute illnesses [3]. In this report, we depict a case of a 31-year-old female who developed a thyroid storm with no apparent underlying risk factors and no obvious inciting events.

## Case presentation

A 31-year-old female with no reported past medical history presented complaining of fever, chills, dizziness, and generalized weakness. The onset of symptoms was acute and started approximately 24 hours prior to arrival to the emergency department. In addition, the patient reported having progressively worsening abdominal discomfort and diarrhea for the last three months. On the physical examination, the patient was febrile with a maximum temperature of 39.7 ℃, hypertensive with a blood pressure of 143/77 mmHg, tachycardic with a heart rate of 141 beats per minute, and had an oxygen saturation of 100% on ambient air. Pertinent physical examination findings included mild disorientation and diffuse abdominal discomfort to palpation. There was no exophthalmos, thyroid mass, thyromegaly, or thyroid tenderness appreciated.

**Figure 1:**
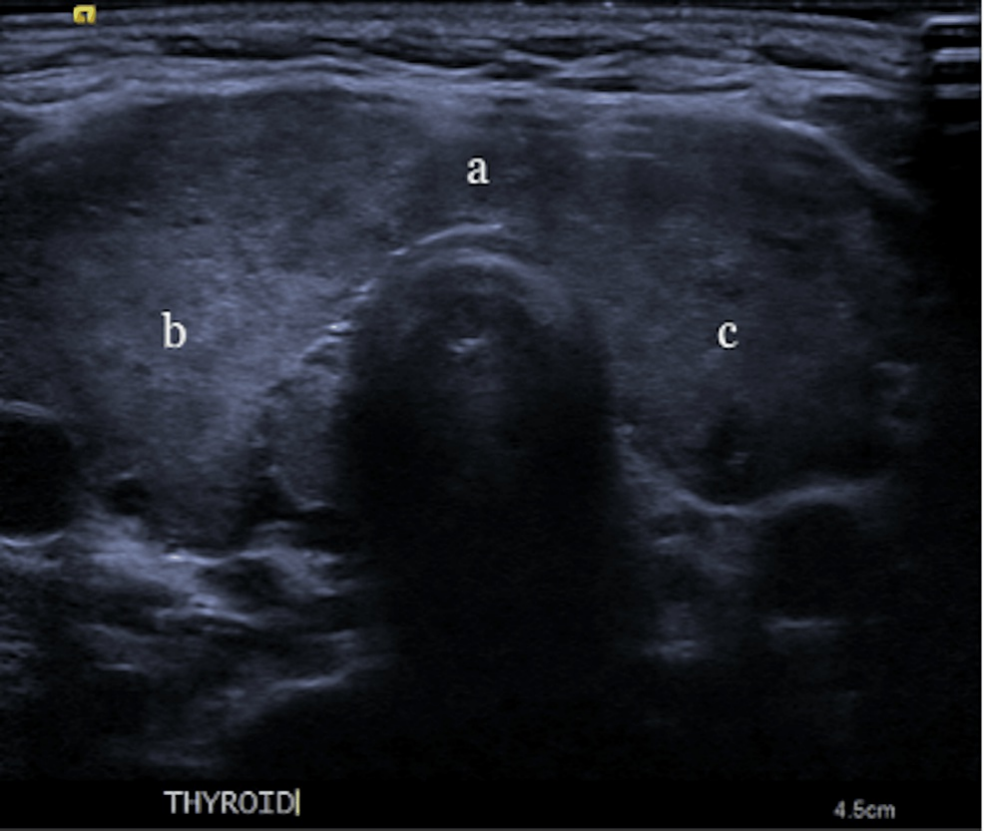
Ultrasound of the thyroid gland Ultrasound of the thyroid gland showing an enlarged and diffusely abnormal-appearing gland with increased echogenicity. The right lobe measures 7.2 x 2.1 x 2.4 cm. The isthmus measures 0.6 cm in thickness. The left lobe measures 6.8 x 1.9 x 2.2 cm. a: Isthmus of the thyroid gland; b: right lobe of the thyroid gland; c: left lobe of the thyroid gland.

**Figure 2:**
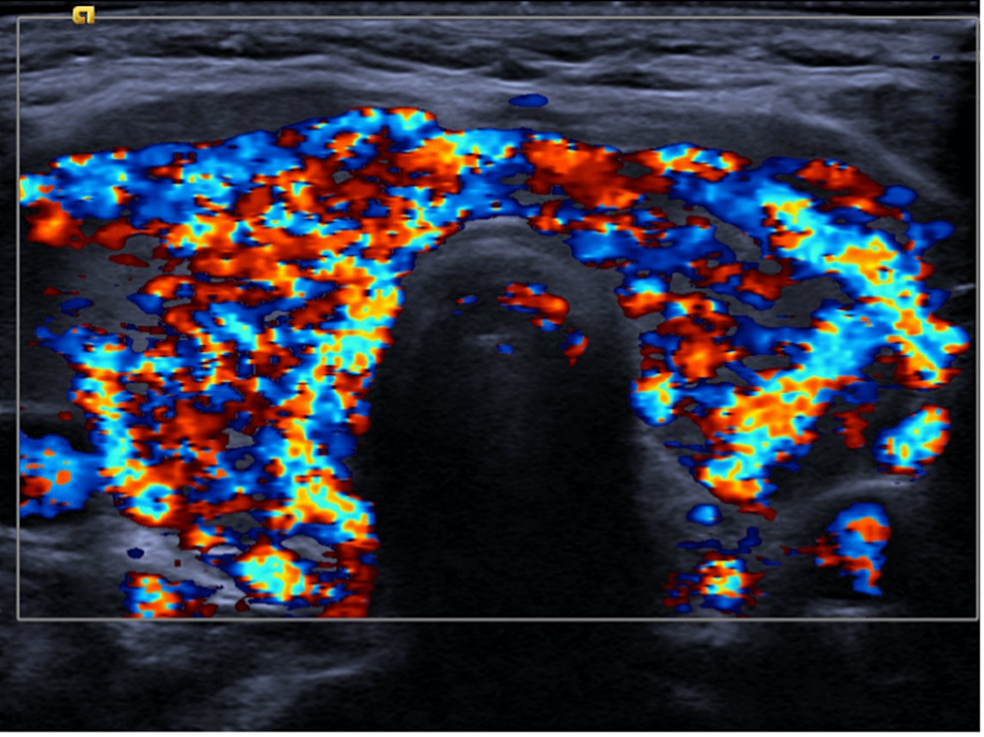
Ultrasound of the thyroid gland with Doppler Ultrasound of the thyroid gland showing increased vascularity on color Doppler consistent with thyroid inferno.

Diagnostic workup was significant for a mild leukocytosis of 12.7 x 10^3^ µL (reference range: 3.5-10.5 × 10^3^ µL), a remarkably low thyroid stimulating hormone of less than 0.005 uIU/mL (reference range: 0.40-4.50 uIU/mL), an elevated free T4 greater than 7.8 ng/dL (reference range 0.8-1.8 ng/dL), T3 uptake less than 0.20 TBI (reference range: 0.8-1.3 TBI), an elevated thyrotropin receptor antibody level of 27.4 IU/L (reference range: 0.00-1.75 IU/L), and an elevated thyroid stimulating immunoglobulin of 24.3 IU/L (reference range: 0.00-0.55 IU/L). Additional workup included an electrocardiogram that showed sinus tachycardia, a transthoracic echocardiogram with a normal left ventricular ejection fraction, no valvular pathology, and no wall motion abnormalities. Computed tomography (CT) scans of the head, abdomen, and pelvis were unremarkable for acute pathology. Laboratory workups, including lactic acid, coagulation factors, hemoglobin, coronavirus disease 2019 (COVID-19) polymerase chain reaction (PCR), and rapid influenza antigens, were all within normal limits. Upon presentation, the patient had a score of 70 on the Burch-Wartofsky Point Scale, which is highly suggestive of thyroid storm. A thyroid ultrasound was obtained and revealed a hypervascular appearance along with increased echogenicity without nodules as depicted in Figure 1 and Figure 2.

Ultimately, the patient was diagnosed with thyrotoxic crisis and immediate treatment was initiated with one dose of 100 mg intravenous (IV) hydrocortisone, 60 mg of propranolol every six hours, 250 mg of propylthiouracil (PTU) every four hours, and potassium iodine supplementation. In addition, 4 grams of cholestyramine given every eight hours was administered to reduce enterohepatic recycling of thyroxine. Throughout the hospitalization, the patient remained hemodynamically stable and made significant clinical improvement. A summary of the free T4 trend is summarized in Table 1.

**Table 1:** Free T4 (thyroxine) level trend during the hospital course Free T4 reference range: 0.8-1.8 ng/dL. Initial treatment with IV hydrocortisone, propranolol, propylthiouracil, potassium iodine supplementation, and cholestyramine was administered on the day of admission.

	Admission	Hospital day 2	Hospital day 3
Free T4 (ng/dL)	>7.8	7.1	6.2

On hospital day 3, the patient was deemed clinically stable for discharge with close endocrine and laboratory follow-up recommended. Discharge medications included 20 mg methimazole taken twice a day, 80 mg propranolol taken three times a day, and 4 grams of cholestyramine taken three times daily.

## Discussion

Thyrotoxicosis, a severe acute life-threatening form, is a rare endocrine emergency that requires emergent intervention. In the United States, the incidence is reported to be 0.57-0.76 cases per 100,000 persons each year and has an estimated mortality rate of 8-25% [4]. Studies have shown that early intervention of thyroid storm can help curtail mortality in patients. Cardinal features of thyroid storm may include fever, tachycardia, hemodynamic instability, and central nervous system (CNS) abnormalities. The Burch-Warsofsky Point Scale serves as a quantitative diagnostic assessment that predicts the severity of thyrotoxicosis using the following variables: thermoregulatory dysfunction; CNS effect; gastrointestinal effect, such as abdominal pain and diarrhea; cardiovascular effect; cardiac failure; and precipitant history. Well-established predisposing factors and triggers of thyroid storm include but are not limited to long-term untreated or undertreated hyperthyroidism, trauma, infection, surgery, childbirth, drug-induced state or reaction, heart failure, and diabetic ketoacidosis [5].

In this case, the patient did not have any apparent precipitating events or underlying risk factors, which is an atypical presentation. The patient had a Burch-Warsofsky Point Scale above 70, which corresponds to a high index of clinical suspicion for a thyrotoxic crisis. Early therapeutic interventions are essential when treating thyrotoxicosis, and multiple studies have demonstrated that optimal management aims to minimize thyroid hormone synthesis and concomitantly circulating hormone levels that ultimately curtails further end-organ damage. Prompt therapy includes administration of steroids, beta blockade therapy, and thioamide agents prior to administration of iodine therapy to prevent the release of precursor thyroid hormones in addition to symptomatic treatment [6]. Steroid therapy, such as IV hydrocortisone or IV dexamethasone, helps prevent the onset of acute adrenal insufficiency in the setting of low adrenal reserves during a thyroid storm. Concomitant administration of non-selective beta blockade therapy decreases the peripheral conversion of T4 to T3, which prevents the impending complications of cardiovascular collapse caused by increased beta receptor activity [7]. Subsequently, thioamides, such as PTU or methimazole, inhibit thyroid peroxidase, thus inhibiting the formation of T3 and T4 from thyroglobulin.

Lastly, it is imperative to inhibit the proteolytic release of preformed thyroid hormones from thyroglobulin via iodine therapy to achieve normalization of thyroid hormones. This particular order of therapeutic interventions is important to follow for the safe and effective management of thyroid storm: blockage of peripheral conversion of T4 → T3 to inhibit the peripheral effects, inhibit thyroid hormone synthesis, and inhibit the release of T3 and T4 from the gland. Compliance to this protocol can help achieve normalization of the acute hyperthyroid state as was accomplished in our patient who presented with neither underlying risk factors nor a previously established diagnosis of hyperthyroidism, which is uncommon.

## Conclusions

Thyrotoxic crisis is a rare and potentially life-threatening hypermetabolic syndrome, clinically characterized by, but not limited to, fever, orbitopathy, marked tachycardia, hypertension, dysrhythmia, high-output heart failure, hyperreflexia, and tremors. The primary predisposing determinants include abrupt discontinuation of antithyroid medication, thyroid surgery, trauma, acute illnesses, parturition, use of iodinated contrast medium, burns, stroke, iatrogenic etiology, and pregnancy. It is crucial to recognize the signs and symptoms of thyrotoxic crisis in patients who experience these types of acute stressors as early detection and appropriate management can reduce potential complications due to thyroid storm and/or the need for surgical intervention. Irrespective of predisposing factors, it is paramount that the clinical manifestations of thyrotoxicosis and thyroid storm be recognized promptly in order to improve clinical outcome and reduce morbidity as patients can present atypically and in the absence of apparent underlying risk factors and triggers.
